# *Rothia mucilaginosa* Meningitis in a Child with Myelodysplastic Syndromes

**DOI:** 10.1155/2021/9946868

**Published:** 2021-09-13

**Authors:** Fumihiro Ochi, Ryota Nakamura, Reiji Miyawaki, Kyoko Moritani, Shinobu Murakami, Hisamichi Tauchi

**Affiliations:** ^1^Department of Pediatrics, Ehime University Graduate School of Medicine, Toon, Ehime, Japan; ^2^Clinical Laboratory Division, Ehime University Hospital, Toon, Ehime, Japan

## Abstract

*Rothia mucilaginosa* is a Gram-positive coccus and an opportunistic pathogen in immunocompromised hosts. The microorganism has been implicated in serious infections, including bacteremia meningitis or endocarditis. However, there is a dearth of investigations on meningitis, especially in children. As this infection is rare and only a few cases have been recorded, evidence-based guidelines for adequate infection treatment are lacking. We herein report the case of a 12-year-old boy with myelodysplastic syndromes (MDS) presenting with a change in mental status who was diagnosed as having febrile neutropenia and bacterial meningitis caused by *R. mucilaginosa* at 23 days after unrelated cord blood transplant. In our case, the minimum inhibitory concentrations (MICs) of meropenem and vancomycin (VCM) were both ≤1 *μ*g/mL, whereas the MIC of daptomycin (DAP) was 4 *μ*g/mL. The patient was treated with intravenous antimicrobial therapy due to meropenem for 43 days because he had febrile neutropenia. During follow-up, the patient had no neurological complications. We retrospectively reviewed the antimicrobial susceptibility of all *R. mucilaginosa* isolates (*n* = 5) from blood or cerebrospinal fluid cultures at our hospital. The MIC of VCM was <0.5 *μ*g/mL for all strains, whereas the MIC of DAP was ≥2 *μ*g/mL for all strains. The MIC of MEPM was >1 *μ*g/mL for one strain. We recommend choosing VCM as the primary treatment for invasive *R. mucilaginosa* infections until antimicrobial susceptibility results are known, especially in immunocompromised children.

## 1. Introduction

*Rothia mucilaginosa*, previously known as *Stomatococcus mucilaginosus*, is a Gram-positive and coagulase-negative coccus occurring as a commensal organism in the oral cavity and respiratory tract [1]. The microorganism is an opportunistic pathogen in immunocompromised patients and has been implicated in several infections, including bacteremia, meningitis, pneumonia, arthritis, endocarditis, and/or osteomyelitis [2, 3]. Although meningitis due to *R. mucilaginosa* is a rare but potentially lethal infection in patients with neutropenia, there is a dearth of investigations on meningitis, especially in children [3, 4]. In addition, because this infection is rare and only a few cases have been recorded, evidence-based guidelines for adequate infection treatment are lacking. Herein, we report a case of *R. mucilaginosa* meningitis in a 12-year-old boy.

## 2. Case Presentation

A 12-year-old boy was admitted to our hospital under the diagnosis of myelodysplastic syndromes (MDS) with monosomy 7. He was treated with an allogeneic bone marrow transplant for MDS after four azacytidine cycles. As engraftment failed, he received an unrelated cord blood transplant (U-CBT). At 23 days after U-CBT, he presented with fever, vomiting, and a change in mental status. Upon physical examination, he was conscious but drowsy (Glasgow Coma Scale score of 13, E3V4M6). Neurological examinations identified a stiff neck and Kernig and Brudzinski signs. He had an aphthous ulcer on the buccal mucosa. His vital signs were as follows: body temperature, 39.7°C; blood pressure, 112/60 mmHg; heart rate, 112 beats/min; respiratory rate, 28 breaths/min; and oxygen saturation, 100% at room air.

**Figure 1:**
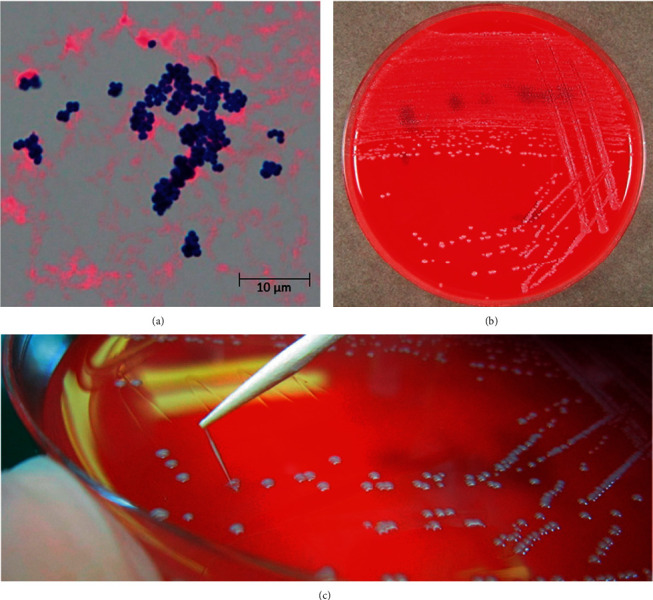
Macroscopic and microscopic appearance of *Rothia mucilaginosa.* (a) Gram staining of characteristic blue cocci from a cerebrospinal fluid culture at 37°C for 24 h. (b) A blood agar plate showing *R. mucilaginosa* colonies in a cerebrospinal fluid sample from the patient, cultured over 24 h at 37°C in CO_2_. (c) Sticky and slimy colonies and a positive string test result.

Laboratory examinations revealed a decreased leukocyte count of <100 cells/*μ*L. The C-reactive protein level was 13.45 mg/dL, and the serum procalcitonin level was 0.79 ng/mL. A lumbar puncture was performed, and his cerebrospinal fluid (CSF) profile revealed 110 leukocytes/*μ*L, with lymphocytic predominance (67%). The CSF total protein level was 76 mg/dL, and the CSF glucose level was 12 mg/dL (CSF glucose/serum glucose = 0.074). Microbiological Gram staining of CSF revealed only typical Gram-positive cluster forming *cocci* (Figure 1(a)). Two rounds of aerobic blood cultures from the peripheral vein and central venous catheter line were negative. As an empirical therapy, we commenced the treatment with meropenem (MEPM; 6 g/day) and vancomycin (VCM; 3 g/day). We observed that Gram-positive colonies were sticky in nature, whitish/gray, nonhemolytic, smooth, and round after 24 h of incubation at 37°C in CO_2_ in sheep blood agar (Figures 1(b) and 1(c)). Stretching of the colonies isolated from our patient's sample resulted in the formation of a string 8 mm in length (with a string ≥5 mm defined as positive). To corroborate this, we confirmed the organism as *R. mucilaginosa* using matrix-assisted laser desorption/ionization mass spectrometry (confidence score, 2.405) at 1 day. *R. mucilaginosa* also grew in an oral aphtha sample.

The minimum inhibitory concentration (MIC) values of the studied antimicrobials were determined by broth microdilution. The MIC for *R. mucilaginosa* was ≤0.06 *μ*g/mL for penicillin *G*, ≤0.25 *μ*g/mL for ampicillin (ABPC), ≤0.25 *μ*g/mL for sulbactam/ampicillin, ≤0.25 *μ*g/mL for tazobactam/piperacillin, 0.5 *μ*g/mL for cefazolin (CEZ), 0.25 *μ*g/mL for ceftriaxone, 1 *μ*g/mL for MEPM, 1 *μ*g/mL for VCM, 1 *μ*g/mL for linezolid, and 4 *μ*g/mL for daptomycin (DAP). We completed the VCM administration on day 3. Although the fever continued, the CSF cultures were negative on day 7. Peripheral blood neutrophil counts had recovered to >500 cells/*μ*L on day 11. Similarly, the fever was alleviated on day 14. In total, we performed antimicrobial treatments due to MEPM therapy for 43 days. During follow-up, the patient remained afebrile and had no neurological complications, including hearing loss.

## 3. Discussion

We present the case of a 12-year-old boy diagnosed as having *R. mucilaginosa* meningitis, MDS, and febrile neutropenia. Bone marrow transplant or chemotherapy in the treatment of MDS induces not only long-term neutropenia but also extensive mucositis. Chavan et al. reported 11 children who developed clinically significant *R. mucilaginosa* infections, including three deaths, directly attributable to the microorganism [5]. Almost all patients had severe neutropenia, central-line catheters, and mucosal breakdown at the time of infection. They described that *R. mucilaginosa* can lead to life-threatening infections in immunocompromised hosts, especially in profoundly neutropenic patients [2, 4, 5]. Our patient had the risk factors of *R. mucilaginosa* infection, such as severe neutropenia, central-line catheter use, and mucosal breakdown at the time of infection, that were reported in previous studies [5, 6].

Recently, high-dose ampicillin, third-generation cephalosporins, rifampin, chloramphenicol, and VCM have been reported to be active in the treatment of invasive *R. mucilaginosa* infections [2, 4, 7, 8]. As *R. mucilaginosa* meningitis is rare and only a few cases have been reported, no guidelines have been established to assist in antibiotic selection for the treatment of invasive *Rothia* infections. Accordingly, the most effective antibiotics for *R. mucilaginosa* meningitis are unknown. Choosing adequate antibiotics is important because antimicrobial agents penetrate the blood-brain barrier less when little or no meningeal inflammation is present in patients with neutropenia. In addition, repeated or prolonged exposure to prophylactic or therapeutic broad-spectrum antibiotics may lead to the selection of pathogenic germs [4]. These factors make *R. mucilaginosa* meningitis treatment more difficult.

Lee et al. described the clinical characteristics of 16 immunocompromised patients who developed meningitis from *R. mucilaginosa* [4]. On the basis of a literature review, they recommended the addition of high-dose ampicillin and rifampin as first-line therapies against *R. mucilaginosa* meningitis. Ramanan et al. also reported that all *R. mucilaginosa* isolates were susceptible to penicillin, MEPM, and VCM, but 33% were oxacillin resistant [2]. However, recently, Kayman et al. reported that isolates from patients with bacteremia were resistant to beta-lactams and susceptible to VCM [9]. Moreover, Getzenberg et al. also reported that the antibiotic susceptibility of *R. mucilaginosa* isolates were 3% for penicillin, 0% for oxacillin, 76% for CEZ, 73% for MEPM, and 100% for VCM [7]. All the cases were successfully treated with VCM. Maraki et al. reviewed 19 published cases (*n* = 20) of *R. mucilaginosa* pneumonia. VCM alone or in combination with other antibiotics have been successfully used for the treatment of *R. mucilaginosa* pneumonia [10]. On the basis of these data, they recommended the administration of VCM as a first-line therapy against invasive *R. mucilaginosa* infection cases.

From now on, owing to the increasing number of reported cases of relative penicillin resistance, other agents are increasingly needed. DAP is used to treat patients with GPC bacteremia or meningitis as empirical therapy. Recently, because *R. mucilaginosa* isolates with high MICs to DAP were observed, caution is required when treating *R. mucilaginosa* infection with DAP. Bruminhent et al. described that the MIC of DAP was 2.0 *μ*g/mL in their case isolate [11]. We retrospectively reviewed the antimicrobial susceptibility of all *R. mucilaginosa* isolates (*n* = 5) from blood or CSF cultures at our hospital between January 2016 and December 2020 (Table 1), revealing that some strains were resistant to *β*-lactam antibiotics, including ABPC. Moreover, the MICs of VCM and LZD were <0.5 *μ*g/mL for all strains, whereas the MIC of DAP was ≥2 *μ*g/mL for all strains. The MIC of MEPM was >1 *μ*g/mL for one strain.

In our case series, the antibiotic susceptibility of *R. mucilaginosa* isolates was 60% for penicillin, 60% for ABPC, 60% for CEZ, 80% for MEPM, and 100% for VCM. The antimicrobial susceptibility results differed among the isolates. Breakthrough GPC bacteremia or meningitis in patients treated with *β*-lactam antibiotics or DAP may reflect emergence of *β*-lactam antibiotics or DAP nonsusceptibility in the infecting isolate.

Different results regarding the antibiotic susceptibility of *R. mucilaginosa* have been reported by various authors [2–11]. However, the reasons for the differences in the antibiotic susceptibility pattern among *R. mucilaginosa* isolates are not clear. Further research is needed to analyze microbiological and clinical features, including underlying disease, antimicrobial treatment, or oral and gastrointestinal flora.

In summary, we recommend that VCM should be chosen as the primary treatment for invasive *R. mucilaginosa* infections until antimicrobial susceptibility results are known, especially in immunocompromised children.

## Figures and Tables

**Table 1:** Clinical features and distribution of the MICs against antimicrobials of *Rothia mucilaginosa* isolated in our hospital.

	Case 1	Case 2	Case 3	Case 4	Case 5^*∗*^
Age/sex	81 y/F	38 y/F	7 y/M	4 y/M	12 y/M
Underlying disease	Lymphoma	AML	Lymphoma	ALL	MDS
Culture source	Blood	Blood	Blood	Blood	CSF
Infection	Sepsis	Sepsis	Sepsis	Sepsis	Meningitis
MIC (*μ*g/mL)					
Penicillin G	≦0.06	>2	>2	≦0.06	≦0.06
Ampicillin	≦0.25	>4	>4	≦0.25	≦0.25
Cefazolin	0.25	>2	>2	0.5	0.5
Ceftriaxone	≦0.12	>2	>2	0.25	0.5
Meropenem	≦0.06	>1	1	0.5	1
Levofloxacin	>4	>4	1	>4	1
Vancomycin	≦1	1	1	1	1
Teicoplanin	≦1	≦0.5	≦0.5	≦0.5	≦0.5
Daptomycin	4	4	2	2	4
Linezolid	1	1	0.5	1	1

^*∗*^Presented case. ALL, acute lymphoid leukemia; AML, acute myeloid leukemia; CSF, cerebrospinal fluid; F, female; M, male; MDS, myelodysplastic syndromes; MIC, minimum inhibitory concentration; y, year.
